# Oral health status in older adults with social security in Mexico City: Latent class analysis

**DOI:** 10.4317/jced.51224

**Published:** 2014-02-01

**Authors:** Sergio Sánchez-García, Erika Heredia-Ponce, Pablo Cruz-Hervert, Teresa Juárez-Cedillo, Ángel Cárdenas-Bahena, Carmen García-Peña

**Affiliations:** 1 Epidemiological Research and Health Services Unit, Aging Sub-unit. Siglo XXI National Medical Center. Mexican Institute for Social Security. Mexico City, Mexico; 2 Department of Public Health and Oral Epidemiology. School of Dentistry. National Autonomous University of Mexico. Mexico City, Mexico; 3 National Institute of Public Health. Cuernavaca, Morelos, Mexico

## Abstract

Objective: To explore the oral health status through a latent class analysis in elderly social security beneficiaries from Southwest Mexico City. Material and Methods: Cross-sectional study of beneficiaries of the State Employee Social Security and Social Services Institute (ISSSTE, in Spanish) and the Mexican Institute of Social Security (IMSS, in Spanish) aged 60 years or older. Oral health conditions such as edentulism, coronal and root caries (DMFT and DFT ≥ 75 percentile), clinical attachment loss (≥ 4 mm), and healthy teeth (≤ 25 percentile) were determined. A latent class analysis (LCA) was performed to classify the oral health status of dentate patients. Results: In total, 336 patients were included (47.9% from the ISSSTE and 52.1% from the IMSS), with an average age of 74.4 (SD = 7.1) years. The 75th percentile of the DMFT = 23 and of the DFT = 2. Of the patients, 77.9% had periodontal disease. The 25th percentile of healthy teeth = 4. A three class model is adequate, with a high classification quality (Entropy = 0.915). The patients were classified as “Edentulous” (15.2%), “Class 1 = Unfavorable” (13.7%), “Class 2 = Somewhat favorable” (10.4%), and “Class 3 = Favorable” (60.7%). Using “Class 3 = Favorable” as a reference, there was an association (OR = 3.4; 95% CI = 1.8-6.4) between being edentulous and being 75 years of age and over, compared with the 60- to 74-year age group. Conclusion: The oral health in elderly social security beneficiaries is not optimal. The probability of becoming edentulous increases with age. A three-class model appropriately classifies the oral health dimensions in the elderly population.

** Key words:**Elderly, Latent class analysis (LCA), oral health, social security, Mexico.

## Introduction

The increase in life expectancy, and particularly global aging, is a successful advance in humanity. Technology innovation in the prevention and cure of many diseases, together with a lower exposure to unsafe conditions should increase the expectations of reaching an appropriate adulthood with better health (1). Of all public health problems that are derived from aging and the expansion of the population, oral health represents a major expenditure for the Health System (2).

The elderly population in Latin America suffers from poor oral health, visits oral healthcare clinics less often, and loses teeth due to poorly controlled chronic diseases and inadequate oral hygiene more than due to the effect of age (3). More than half of the elderly population in Mexico receives professional dental attention once per year. These follow-ups are usually attended in the private sector because the State and the Social Security System do not provide elderly individuals the proper care (4). Of the population between 60 and 64 years of age, 17.2% are edentulous. This percentage increases with age, and after 85 years of age, 50.5% of patients have lost all their teeth (2). Poor oral hygiene and deficient nutrition are characteristic of a poor lifestyle and are considered etiopathological factors of oral morbidity in the elderly (5).

Caries and periodontal disease are the main causes of tooth decay (6,7). Partial or total tooth loss has an impact on food swallowing, sound pronunciation, and strength and muscular activity. In addition, food deprivation negatively affects the patient’s general health and quality of life (8-10). Dental prosthesis rehabilitation substantially improves the personal appearance and quality of life (11). However, the perception of elderly patients regarding the necessity of dental prostheses reportedly differs from that of the oral health care practitioner (12).

Therefore, the aim of the present study was to explore the oral health status of elderly beneficiaries of the ISSSTE and IMSS of Southwest Mexico City through a latent class analysis.

## Material and Methods

A cross-sectional study was performed between January and March of 2010. A representative sample of elderly beneficiaries of the primary healthcare services of the Family Medicine Clinic of the ISSSTE and the Family Medicine Unit of the Southwest Mexico City IMSS was analyzed. The selection criterion for these units was the similar geographical area.

The sample included 200 beneficiaries (age 60 years or older) of primary care at the ISSSTE and IMSS. The patients were selected randomly using the databases of elderly patients who had required assistance during the months of January and July of 2009. Patients who refused to participate, who could not be contacted at the registered address and telephone number, or who had died were excluded from the study.

- Data collection

All patients were contacted via telephone or by letter at their registered address and were invited to participate in the study. The patients confirmed their agreement to participate by verbal and signed consent.

The interview and oral evaluation were performed in their respective clinic or unit.

The aim of the interview was to obtain information on the subjects’ sociodemographic characteristics (i.e., sex, age, marital status, schooling, paid employment, and living situation), chronic diseases, cognitive function, and depressive symptoms.

The information on chronic degenerative diseases was obtained by asking the patient whether a physician had diagnosed them with any disease. A positive answer led to an interrogation regarding the diagnosis of cancer, diabetes mellitus, dyslipidemias, gout, hypertension, heart attack, cerebrovascular conditions, chronic obstructive pulmonary disease, mental disorders, cirrhosis of the liver, biliary or renal lithiasis, gastric or duodenal ulcer, chronic renal failure, prostate hyperplasia, hip or femur fracture, other fractures, and any disease of more than 3 months evolution.

Cognitive function was assessed with the mini-mental state evaluation (MMSE). Cognitive impairment was indicated by a score of ≤ 23 points, adjusted by age (13).

Depression was measured using the 30-item Geriatric Depression Scale (GDS) (14). Scores from 0 to 9 points indicated a “Normal” status (no depressive symptoms), 10 to 19 points indicated “Moderate depression,” and 20 to 30 points indicated “Severe depression” (15).

The oral evaluation analyzed the oral health status (edentulism, history of coronal or root caries, clinical attachment loss, and healthy teeth) and the dental prostheses (use, function, and necessity of replacement).

A previously trained and standardized dental surgeon performed the assessment (Kappa ≥ 0.80) in accordance with the World Health Organization (WHO) criteria to assess dental status and attachment loss in the periodontal tissues (16). The oral evaluation was performed with the patient siting on a chair (or when necessary, on a wheel chair), under natural light, using a No. 5 mirror and a WHO-type periodontal probe (PCP 11.5B, Hu–Friedy). Coronal caries (DMFT index, the number of Decayed⁄Missing⁄Filled Teeth), and root caries experience (DFT index, the number of Decayed/Filled roots).

The 75th percentile of the DMFT and DFT indexes in the elderly patients who participated in the study was established to define a cut-off point for the presence of coronal and root caries. The cut-off point to classify the elderly patients with a high caries index was ≥ 23 teeth in the DMFT index and ≥ 2 roots in the DFT index. The 25th percentile corresponded to a low number of healthy teeth, and the cut-off point was ≤ 4.

Clinical attachment loss is the predominant clinical manifestation and main determinant of periodontal disease. It was considered when one or more sites had an attachment loss of ≥ 4.0 mm.

**Figure 1:**
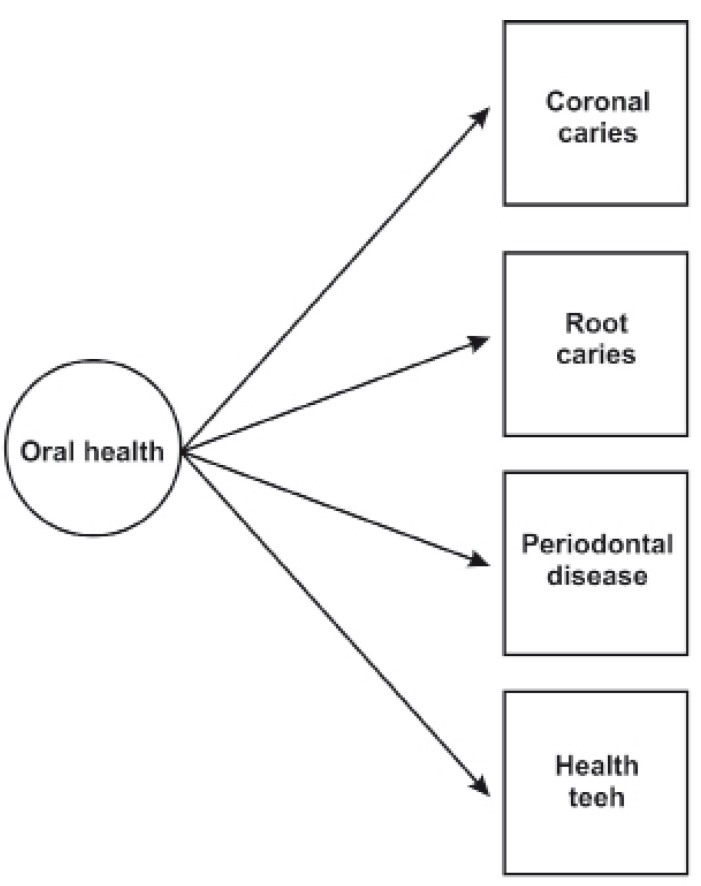
Latent class model for oral health in an elderly dentate population.

Oral health was considered a latent variable in dentate elderly patients because it is an integral variable that cannot be observed but must be inferred and measured from a group of observable and explanatory variables (i.e., dental and root caries history, clinical attachment loss, and healthy teeth) (Fig. 1). A latent class analysis was used because it is a statistical technique that studies the existence of one or more latent variables inferred from a group of observable and explanatory variables and defines, based on its classes, a classification or typology of the individuals in the sample (17).

The investigation protocol was reviewed and approved by the National Health Research Committee and the IMSS Research Ethics Committee (Register No. 2005-785-170).

- Statistical analysis

The descriptive statistical analysis and the percentage distribution were calculated using IBM SPSS (Statistical Package for Social Science) version 19.0 for Windows. The Mann-Whitney U test was used to determine differences between the two groups, and Pearson’s Chi Square test was used to determine the uniformity of the frequency and distribution. A multinomial logistic regression analysis was performed to determine the strength of the association (Odds Ratio, OR; 95% Confidence Interval, 95% CI) between the oral health classification and the sociodemographic variables (i.e., sex, age, marital status, schooling, paid employment, and living situation), as well as the chronic health conditions, cognitive function, and depressive symptoms.

A latent class model analysis was performed using the Mplus version 5. The latent class analysis (LCA) is an approach that classifies the individuals into groups based on the patterns of individual response. The purpose of using the LCA in this study was to distinguish the optimal number of classes that characterize the oral health of the elderly beneficiaries of the IMSS and ISSSTE. The choice of a model in terms of the classes for oral health was determined by progressively increasing its number and contrasting the results with each subsequent model using the Lo-Mendell-Rubin test, in which a nonsignificant probability (p > 0.05) suggests that the previous model (with a lower number of classes) is preferable. To assess the goodness of fit of the model, a combination of the Akaike Information Criterion (AIC) and the Bayesian Information Criterion (BIC) were used, in addition to the entropy values, to obtain more information on the model adjustment. Although there is no standard threshold to evaluate entropy, values near 1.0 are desirable (18). Finally, the models were interpreted in terms of their theoretical and practical coherence, with the simplest and most parsimonious model subsequently selected.

## Results

Of the 400 selected elderly patients, 84.0% (n = 336) participated in the study (ISSSTE 47.9%, n=161; IMSS 52.1%, n=175). Of the remaining patients, 2.5% (n = 10) refused to participate, 12.7% (n = 51) could not be located at the registered address or telephone number, 0.5% (n = 2) had died, and 0.3% (n = 1) refused to be clinically evaluated.

Of the total sample (n = 336), 69% (n = 232) were women with a mean age (SD) of 71.4 (7.8) years, and 31.0% (n = 104) were men with a mean age of 71.4 (8.0) years; 44.9% (n = 151) were married, 31.8% (n = 107) were single, and 23.2% (n = 78) were widowers; 5.4% (n = 18) had no school education, 27.1% (n= 91) had attended school for 1 to 6 years, and 67.6% (n = 227) had attended school for 7 or more years; 25.3% (n = 85) had paid employment, 35.1% (n = 118) had unpaid employment, 39.6% (n = 133) were retired, and 19.9% (n = 67) lived by themselves; 7.1% (n = 24) did not present any chronic disease, 37.2% (n = 125) presented one chronic disease, 35.1% (n = 118) presented two chronic diseases, and 20.5% (n = 69) had three or more chronic diseases; 27.4% (n = 92) had mild cognitive impairment, and 29.8% (n = 100) had depressive symptoms.  Table 1 lists the frequency and percentage distribution of the characteristics of the elderly subjects. A statistically significant difference (p < 0.05) was found between the ISSSTE and the IMSS in the frequency and distribution of the variables sex, marital status, and chronic health conditions.

**Table 1:**
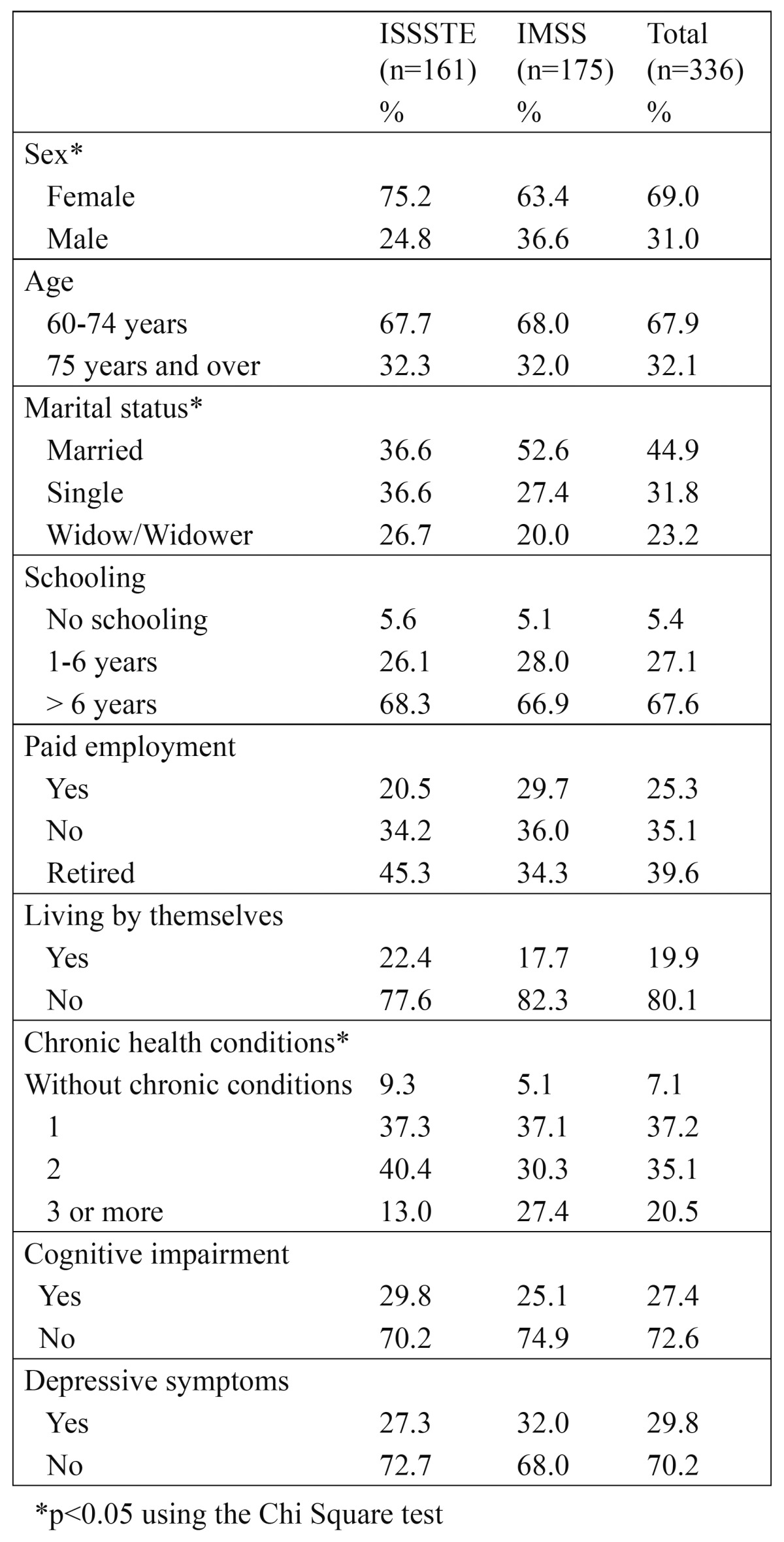
Characteristics of the sample of elderly beneficiaries of the ISSSTE and IMSS.

 Table 2 lists the components of the oral health status (i.e., edentulism, history of dental and root caries, clinical attachment loss, and healthy teeth) of the elderly population in the study. There were statistically significant differences (p < 0.05) in the DMFT and DFT between the ISSSTE and the IMSS.

**Table 2:**
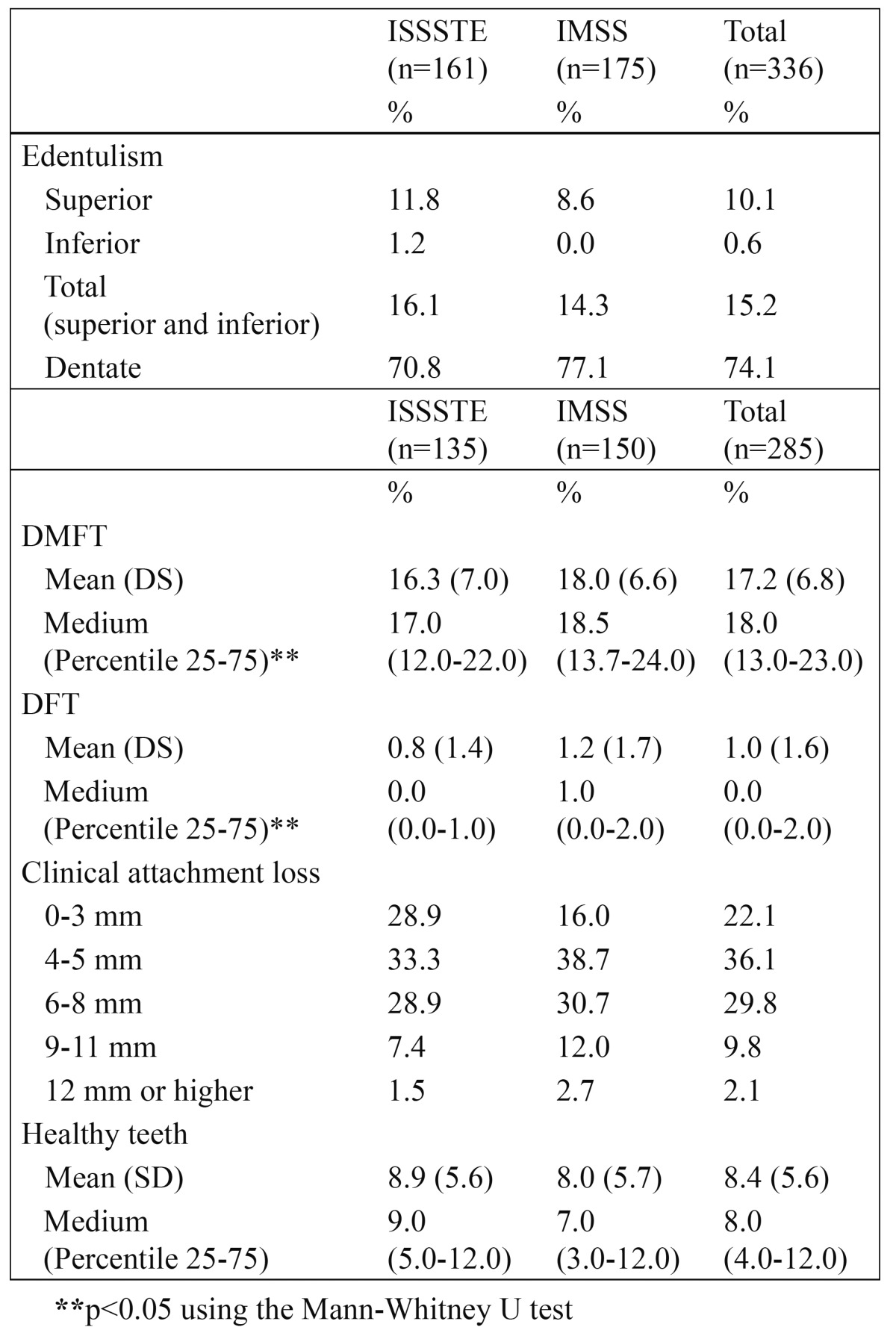
Components of the oral health status of the sample of elderly beneficiaries of the ISSSTE and IMSS.

The LCA revealed three classes for the oral health status of the elderly dentate patients, classified as follows: Class 1 “Unfavorable” (16.1%, n = 46), Class 2 “Somewhat favorable” (12.2%, n = 35), and Class 3 “Favorable” (71.6%, n = 204).  Table 3 lists the conditional probabilities of each oral health component in the elderly dentate patients for each of the three classes, as well as the goodness of fit of the model that presented a high classification quality (Entropy = 0.915).

**Table 3:**
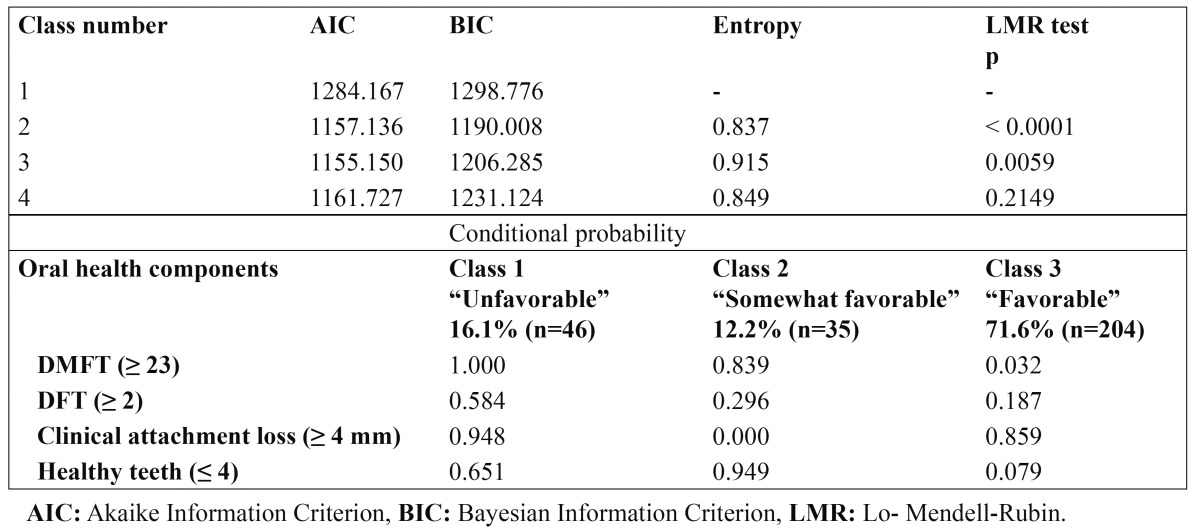
Goodness of fit of the model and conditional probability associated with the latent class of oral health for an elderly dentate population.

The 336 elderly persons were classified as follows: 15.2% (n = 51) as “Edentulous”, 13.7% (n = 46) as “Unfavorable” (Class 1), 10.4% (n = 35) as “Somewhat favorable” (Class 2), and 60.7% (n = 204) as “Favorable” (Class 3). The frequency and distribution of the edentulous and oral health latent classes are presented in  Table 4, together with the sociodemographic variables, chronic health conditions, cognitive function, and depressive symptoms. There was a difference in the frequency and distribution of the edentulous and oral health latent classes between the 60- to 74-year age group and those 75 years of age and over (Pearson’s Chi square = 16.273; DF = 3; p = 0.001). When the strength of association was assessed through a multinomial logistic regression analysis, using the elderly patients with favorable oral health condition as a reference, an OR = 3.4 (1.8 - 6.4) of being edentulous in the elderly patients who were 75 years of age and older was determined, compared with the 60- to 74-year age group. Statistically significant associations were not observed in the unfavorable and somewhat favorable groups (p > 0.05). Moreover, there was no difference between the frequency and distribution of the edentulous patients and the oral health classes with the rest of the study variables (p > 0.05).

**Table 4:**
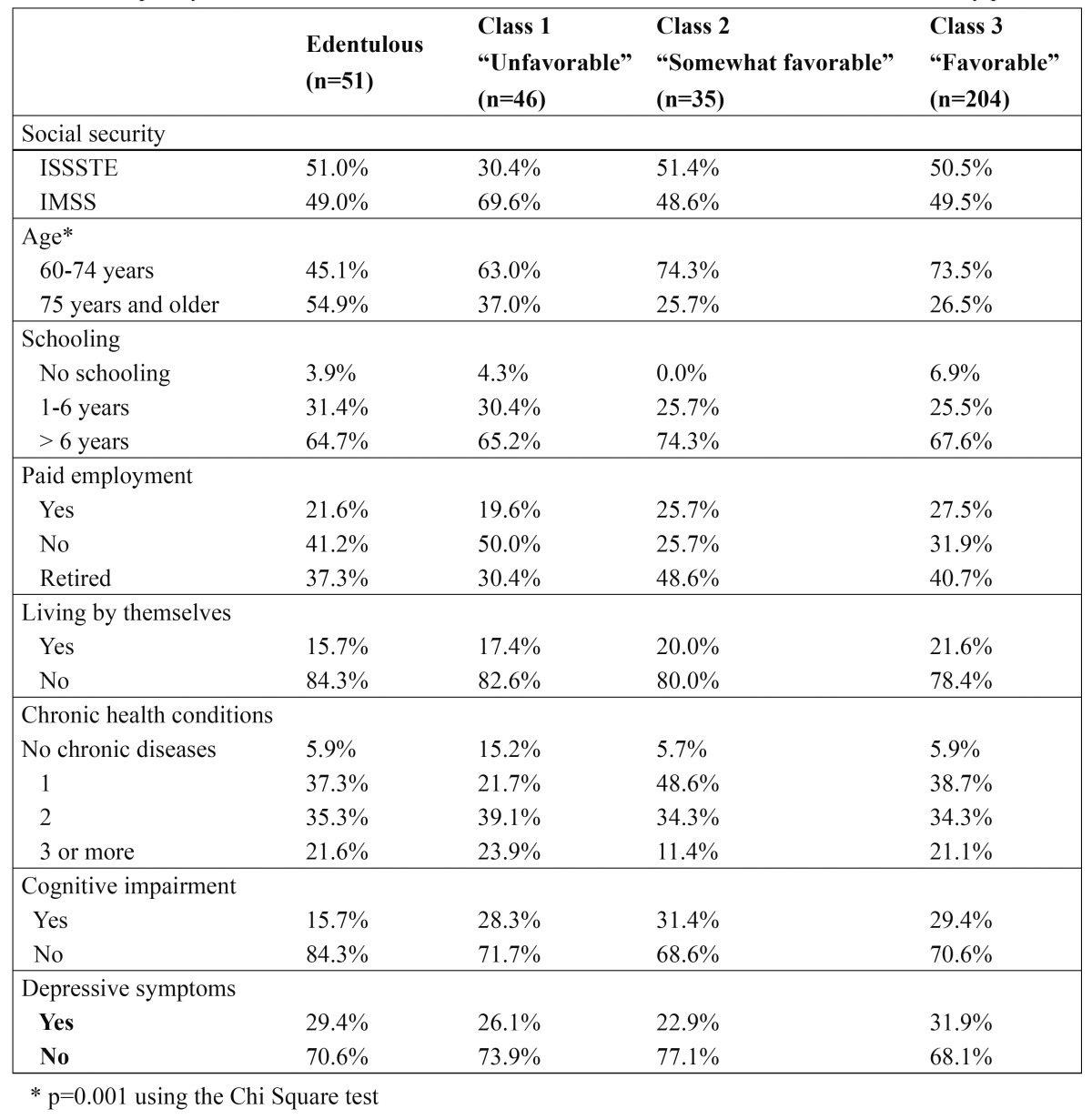
Frequency and distribution of the latent classes in the oral health and edentulism of elderly patients.

## Discussion

Oral health problems begin at an early age and become a greater problem at advanced ages. Such is the case with tooth loss, edentulism, clinical attachment loss, coronal and root caries, oral mucosal lesions, use of nonfunctional dental prostheses (partial or total), low salivary flux, chewing problems, among other conditions (19). Traditionally, the oral health of the population, and particularly of elderly persons, has been limited to clinical indicators and oral indexes, as well as to the presence or absence of a separate disease (20)—that is, each factor separately contributes information on each oral health component, and thus the individuals are not classified based on the patterns of the oral health components.

Using the information regarding the DMFT and DFT indexes, clinical attachment loss, and the number of healthy teeth, an LCA was performed to characterize the oral health in the elderly population. We obtained three classes that can characterize the oral health of elderly dentate persons. Class 1 was characterized by having a higher probability of coronal caries and clinical attachment loss, a medium probability of root caries, and a low number of healthy teeth. Based on the above description, this class was considered to have an unfavorable oral health status. Class 2 was characterized by having a high probability of many coronal caries, a low number of healthy teeth, a low probability of many root caries, and no probability of periodontal disease. Considering the above description, these patients had somewhat favorable oral health. In Class 3, a high probability of clinical attachment loss, a low probability of root and coronal caries, and a low number of healthy teeth were observed. Consequently, this class was considered to have favorable oral health but not optimum due to high probability of clinical attachment loss. The LCA did not consider toothless elderly patients because these individuals comprise a separate group. The LCA helps to characterize oral health and may be a heuristic tool that enables new inferences in the oral health status of the elderly population.

In latent class analysis, sociodemographic characteristics, chronic diseases, cognitive function and depressive symptoms were not considered because this was an exploratory analysis. Further studies should consider the effect of these and other variables in the classification of oral health status in older adults.

The dental status was a serious oral health problem for the elderly in this study and confirmed the reported literature (7). When comparing the DMFT and DFT indexes between the ISSSTE and IMSS, we observed a significant difference, with the IMSS having the higher values in both indexes. Unfortunately, little information is available regarding the oral health status of the Mexican population, and particularly, of the elderly. National Health and Nutrition Surveys do not deem oral health to be an element that should be evaluated, despite reports published on the association of oral and systemic diseases, such as endocarditis, cerebrovascular disease, coronary disease, infarction, hypertension, diabetes mellitus, respiratory disease, and osteoporosis, as well as low birth weight, premature births, nutritional deficiencies, and cancer (21). The only document in Mexico is the 1996-2000 National Survey on Dental Caries and Fluorosis that was performed on children aged 6 to 12 years. The care requirements and the oral health status of the elderly population in Mexico are among the short- and mid-term challenges, serving as a basis for strategy implementation and for a continuous assessment to maintain or improve their oral health (22).

The ultimate result of dental caries and periodontal disease is tooth decay (6,7), which also poses serious implications in the overall health and quality of life in the elderly (8). As previously described, we observed that a total loss of teeth (edentulism) is associated with age (2,19,23).

Notably, the present study involved elderly patients who used health services within a given period and does not represent the totality of elderly beneficiaries in Southwest Mexico City. However, our investigation allows for exploring the oral health conditions of patients using the health services and for planning new studies to answer the unknown factors that cannot be solved in a cross-sectioned study.

Tooth loss, as well as the effects of periodontal disease (attachment loss) and coronal and root caries, must not be considered an inevitable consequence of aging (3,4). Consequently, edentulism and oral health conditions in the elderly population of the present study are the reflection of the lack of public policies with an impact on the oral health of this population in Mexico during the last 6 decades. Indeed, Social Security oral health services provide care at the primary level and are limited to preventive treatment and control, as well as amalgams or composite restorations. All restorative and rehabilitative treatments should be covered by private oral health services (4). Thus, health policies must be implemented to cover this unsolved need (3,4,21).

Another important aspect that has not been considered in the health policies in Mexico is the incorporation of formation programs in undergraduate and graduate studies to address the oral healthcare needs of elderly patients. An inconsistency between what is being taught in the universities and the actual requirements for improving the oral health of the population has been noted (21). Likewise, continuing education programs aimed at oral health professionals must be implemented to address the needs of this population group.

Finally, we conclude that the oral health status of elderly beneficiaries of the ISSSTE and IMSS is similar but not optimal because only 60.7% had a favorable oral health status according to the classification from the oral health LCA that was performed. Furthermore, total tooth loss (edentulism) is associated with age; the older the individual, the more likely he or she is to be edentulous.

## References

[1] Naciones Unidas UN (1991). Resoluciones 46/91 de la Asamblea General de las Naciones Unidas. New York UN.

[2] Medina-Solís CE, Pérez-Núñez R, Maupomé G, Avila-Burgos L, Pontigo-Loyola AP, Patiño-Marín N (2008). National survey on edentulism and its geographic distribution, among Mexicans 18 years of age and older (with emphasis in WHO age groups). J Oral Rehabil.

[3] Marino R (1994). Oral health of the elderly: reality, myth, and perspective. Bull Pan Am Health Organ.

[4] Sánchez-García S, de la Fuente-Hernández J, Juárez-Cedillo T, Mendoza JM, Reyes-Morales H, Solórzano-Santos F (2007). Oral health service utilization by elderly beneficiaries of the Mexican Institute of Social Security in México city. BMC Health Serv Res.

[5] Wilczyńska-Borawska M, Małyszko J, Cylwik-Rokicka D, Myśliwiec M (2012). Prosthetic status and treatment needs for lost masticatory function in haemodialysis patients. Arch Med Sci.

[6] Ong G (1998). Periodontal disease and tooth loos. Int Dent J.

[7] Saunders RH, Meyerowitz C (2005). Dental caries in older adults. Dent Clin North Am.

[8] Mack F, Schwahn C, Feine JS, Mundt T, Bernhardt O, John U (2005). The impact of tooth loss on general health related to quality of life among elderly Pomeranians: results from the study of health in Pomerania (SHIP-O). Int J Prosthodont.

[9] Pallegedara C, Ekanayake L (2008). Effect of tooth loss and denture status on oral health-related quality of life of older individuals from Sri Lanka. Community Dent Health.

[10] Gerritsen AE, Allen PF, Witter DJ, Bronkhorst EM, Creugers NH (2010). Tooth loss and oral health-related quality of life: a systematic review and meta-analysis. Health Qual Life Outcomes.

[11] Ellis JS, Pelekis ND, Thomason JM (2007). Conventional rehabilitation of edentulous patients: the impact on oral health-related quality of life and patient satisfaction. J Prosthodont.

[12] Colussi CF, De Freitas SF, Calvo MC (2009). The prosthetic need WHO index: a comparison between self-perception and professional assessment in an elderly population. Gerodontology.

[13] Reyes-Beaman S, Beaman PE, García-Peña C (2004). Validation of a modified versión of the Minimental State Examination (MMSE) in Spanish. Aging Neuropsychol Cognition.

[14] Yesavage JA, Brink TL, Rose TL, Lum O, Huang V, Adey M (1982). Development and validation of a geriatric depression screening scale: a preliminary report. J Psychiatr Res.

[15] Sánchez-García S, Juárez-Cedillo T, García-González JJ, Espinel-Bermúdez C, Gallo JJ, Wagner FA (2008). Usefulness of two instruments in assessing depression among elderly Mexicans. Salud Publica de Mex.

[16] Sánchez-García S, Reyes-Morales H, Juárez-Cedillo T, Espinel-Bermúdez C, Solórzano-Santos F, García-Peña C (2011). A prediction model for root caries in an elderly population. Community Dent Oral Epidemiol.

[17] Henderson J, Granell R, Heron J, Sherriff A, Simpson A, Woodcock A (2008). Associations of wheezing phenotypes in the first 6 years of life with atopy, lung function and airway responsiveness in mid- childhood. Thorax.

[18] Kendzor DE Caughy MO, Owen MT (2012). Family income trajectory during childhood is associated with adiposity in adolescence: a latent class growth analysis. BMC Public Health.

[19] Petersen PE, Kandelman D, Arpin S, Ogawa H (2010). Global oral health of older people–call for public health action. Community Dent Health.

[20] Sánchez-García S, Juárez-Cedillo T, Reyes-Morales H, De la Fuente-Hernández J, Solórzano-Santos F, García-Peña C (2007). Estado de la dentición y sus efectos en la capacidad de los ancianos para desempe-ar sus actividades habituales [Dental status and its effect on the capacity of the elderly to perform normal activities]. Salud Publica Mex.

[21] Medina-Solís CE, Maupomé G, Avila-Burgos L (2006). Políticas de salud bucal en México: Disminuir una de las principales enfermedades. Una descripción Oral health policies in Mexico: Decreasing one of the main diseases. A description. Rev Biomédica.

[22] Weening-Verbree L, Huisman-de Waal G, van Dusseldorp L, van Achterberg T, Schoonhoven L (2013). Oral health care in older people in long term care facilities: A systematic review of implementation strategies. Int J Nurs Stud.

[23] Polzer I, Schimmel M, Muller F, Biffar R (2010). Edentulism as part of the general health problems of elderly adults. Int Dent J.

